# Giant primary angiosarcoma of an adolescent girl’s breast diagnosed postmortem: a case report

**DOI:** 10.1186/s13256-020-02403-y

**Published:** 2020-06-27

**Authors:** Tchin Darré, Luc Valère Codjo Brun, Falilath Seidou, Toukilnan Djiwa, Marie Claire Ballé, Gado Napo-Koura

**Affiliations:** 1 Department of Pathology, University Teaching Hospital of Lomé, Lomé, BP 1515 Togo; 2 Department of Pathology, University Teaching Hospital of Parakou, Cotonou, Bénin

**Keywords:** Primary angiosarcoma, Breast, Adolescent girls, Postmortem, Sub-Saharan Africa

## Abstract

**Background:**

Angiosarcoma is an endothelial malignant tumor; angiosarcoma located in the breast is extremely rare, less than 0.01%. We report a case of a giant angiosarcoma diagnosed postmortem in a 16-year-old girl in a resource-constrained country.

**Case presentation:**

A 16-year-old black African girl was admitted for altered consciousness and a left breast tumor. She was admitted in a state of apparent death. Her death was confirmed on clinical examination. A postmortem biopsy of the mammary tumor showed standard histology of a proliferation of fusiform or rounded tumor cells in places, which were not very cohesive with marked anisokaryosis and numerous foci of tumor necrosis. Immunohistochemistry showed a positivity of CD31 and factor VIII markers with a proliferation index (Ki-67) estimated at 30%.

**Conclusion:**

Primary angiosarcoma of the breast is exceptional in adolescents and has a poor prognosis, especially in countries with limited resources.

## Introduction

Breast sarcomas represent less than 1% of breast tumor cases and less than 5% of sarcomas [1]. Angiosarcomas are rare vascular cancers; they represent 2 to 3% of soft tissue sarcomas but with strong aggressive and metastatic potential [1]. Angiosarcomas are tumors of young adults; breast localization is rarely described in the literature [2, 3]. Angiosarcomas represent less than 1% of all cases of breast cancer but with rapid progression they can reach a significant size, leading to death in the absence of early and adequate management [4]. Angiosarcoma of the breast is an extremely rare tumor in adolescent girls [1]. Median recurrence-free survival is less than 3 years. The overall 5-year survival is 46% for primary angiosarcoma of the breast and 69% for secondary angiosarcoma [5]. We report a case of giant angiosarcoma diagnosed postmortem in a 16-year-old girl in a resource-constrained country. We detail the epidemiology, morphological, and prognostic aspects of this rare tumor.

## Case presentation

A 16-year-old black African girl was admitted to the emergency room of the Parakou University Hospital Center for altered consciousness and tumor of the left breast. The tumor had been progressing for approximately 6 months before admission, according to her parents. Our patient did not smoke tobacco or drink alcohol. Her medical, surgical, and psychosocial history was unremarkable. She had normal pubertal development. Her parents are of low socio-economic status. The onset of symptoms was marked by the appearance of a small nodule in her left breast, and an evolution marked by a rapid increase in the volume of the tumor, which motivated traditional herbal treatments of unknown nature. No notion of radiotherapy or previous chemotherapy was reported.

Our patient was admitted in a state of apparent death. She had no blood pressure or pulse. Her temperature was 35 °C. She had an estimated Glasgow Coma Scale of 3. She was pronounced dead on clinical examination 10 minutes after admission. Blood samples for laboratory tests of hepatic and renal function and serological tests could not be taken before she died. The parents claimed to have no knowledge of breast cancer in their family. The parents claimed to have consulted two traditional healers and an herbal treatment of unknown nature was administered to the girl, but without improving her health.

**Fig. 1:**
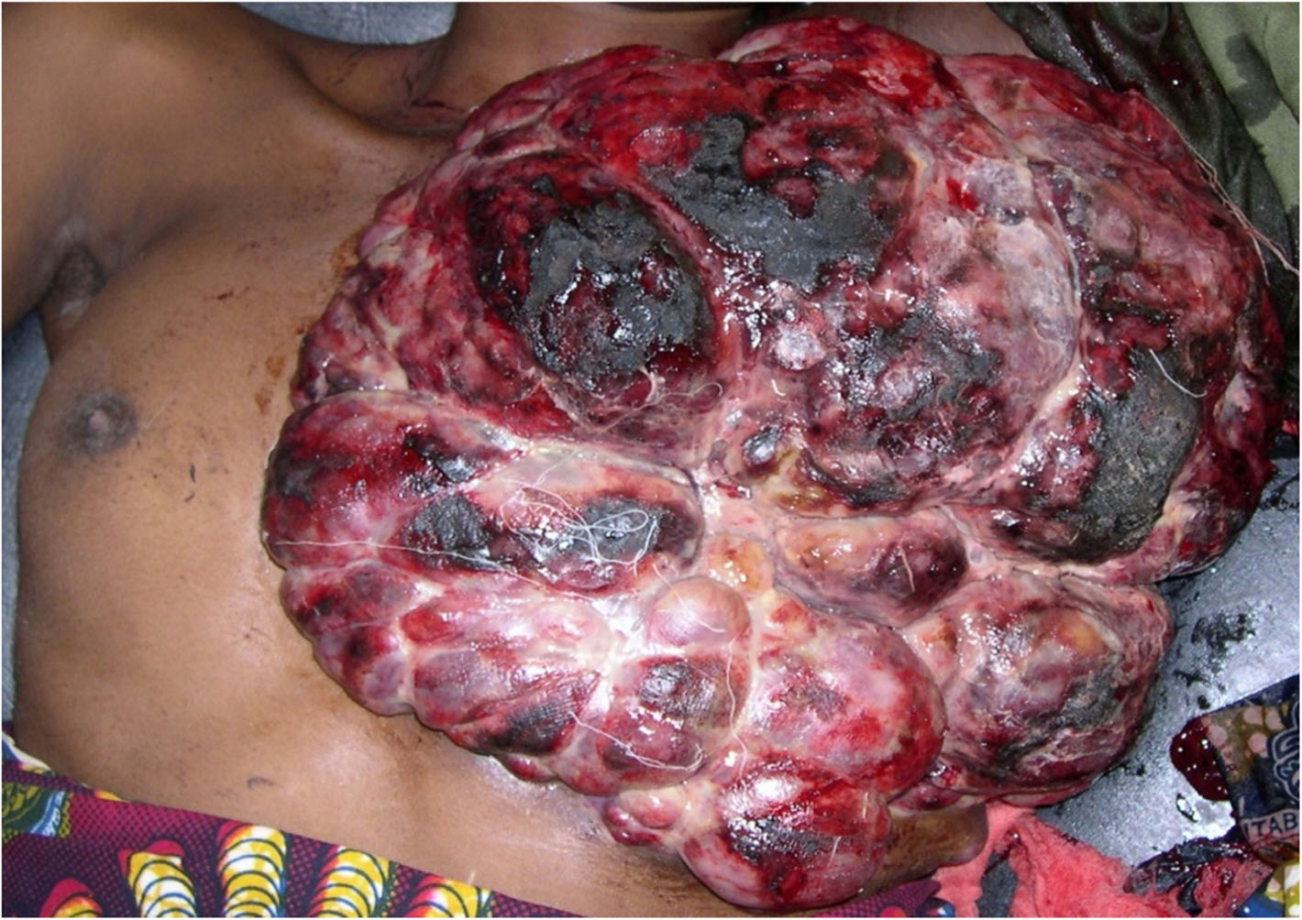
Macroscopy of multinodular and hemorrhagic tumor mass of the left breast

**Fig. 2:**
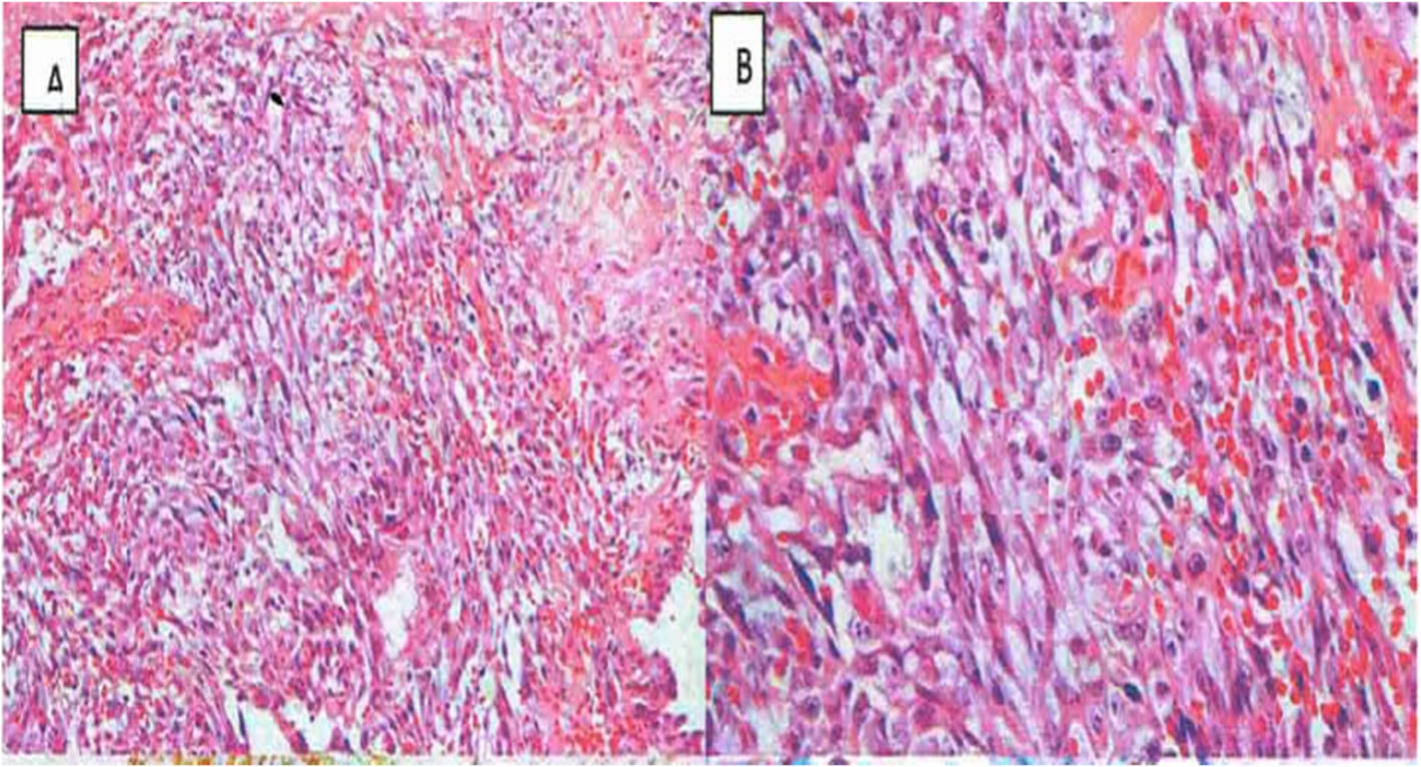
Photomicrograph (original magnification × 10 (**a**) and × 40 (**b**); hematoxylin and eosin stain): Note the presence of anarchic vascular elements

A postmortem examination of the body showed cachexia and a bulky, multinodular, exulcerated, blackish red hemorrhagic mass of the left breast, 35 cm in circumference, partially overflowing on the right hemithorax (Fig. 1). A biopsy sample of the tumor mass was carried out postmortem. Histopathological examination after staining with hematoxylin and eosin showed tumor proliferation made up of anastomosed vascular networks. The tumor cells were spindle shaped, oval, or rounded in places and were not very cohesive. There was a marked anisokaryosis and numerous foci of tumor necrosis (Fig. 2).

**Fig. 3:**
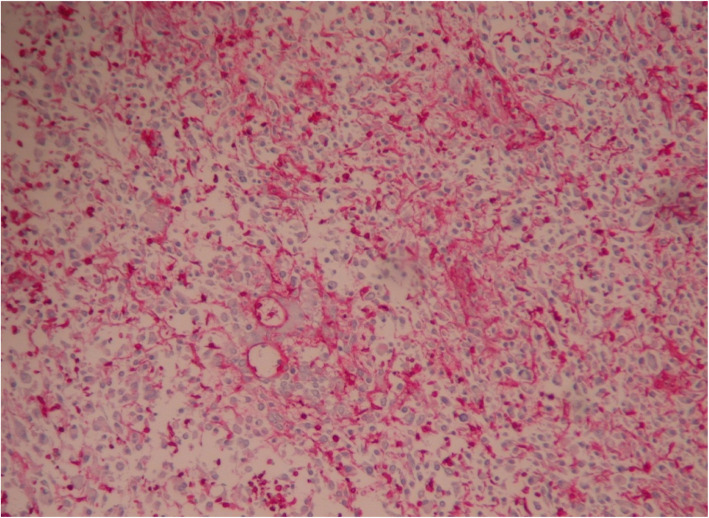
Positive vimentin (factor VIII) staining of angiosarcoma

**Fig. 4:**
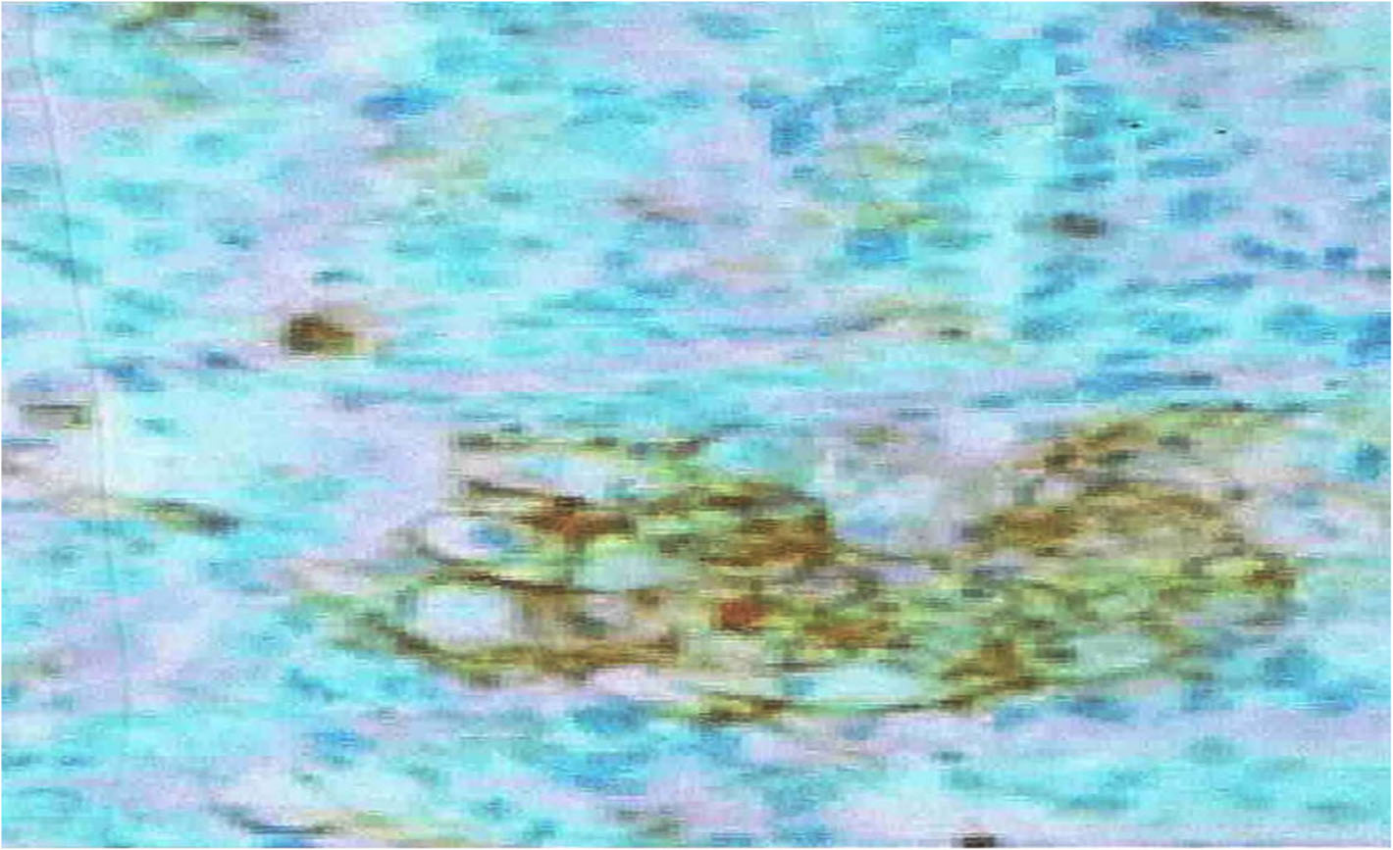
Positive CD31 markers staining of angiosarcoma

The result of a immunohistochemical analysis was positive for CD31 and factor VIII (Figs. 3 and 4). The proliferation index (Ki-67) was estimated at 30%. The expression of the tumor cells was negative for smooth muscle actin (SMA), desmin, melanin A, and S-100. A diagnosis of grade 3 angiosarcoma according to The National Federation of Centers of Cancer Control (FNCLCC) was confirmed.

## Discussion

### Epidemiology

We have reported a case of primary angiosarcoma of the breast in an adolescent girl, received in the emergency room in a state of apparent death and whose diagnosis was made postmortem. This is an extremely rare histological type of cancer that is often seen in the elderly; it has a very poor prognosis. In addition to this rarity and gloomy prognosis of adolescent angiosarcoma, is, in our case, a lack of adequate health policy for cancer care in countries with limited resources.

Angiosarcomas represent a particular form of sarcomas developed at the expense of endothelial cells of the blood and lymphatic vessels and represent 2 to 3% of soft tissue sarcomas but with a strong aggressive and metastatic potential [1, 2]. They can occur in different places and in different organs and can have different characteristics. Approximately 50% of angiosarcomas develop in the head and/or neck [4]. Breast angiosarcoma is a rare location [5]. It comes in two distinct forms: primary, which appears in the breast parenchyma of young women between 20 and 40 years, with no history; and secondary, which develops in the skin, the chest wall, or the breast parenchyma after surgery and postoperative radiotherapy for breast cancer [5]. Its prevalence in primary malignant breast tumors is 0.04% and in mammary sarcomas 8% [1]. This proliferation represents 0.3% of all pediatric sarcomas and is extremely rare in adolescents, where it represents 10% of angiosarcomas [5].

### Clinical

The clinical presentation of breast angiosarcomas differs little from that of a “classic” breast carcinoma. It is usually a painless breast mass [1]. It is necessary to know how to evoke this diagnosis by noting elements such as a large size of the lesion up to 19 cm in diameter, rapid growth, and the almost constant association with an abnormality of the skin in the form of a reddish, purplish, even pulsating coloring and sometimes of skin ulceration [5]. Our patient presented a large tumor with a 35 cm long axis. Distant metastases can be found, but lymph node involvement is exceptional [1]. Cachexia is a sign often associated with cancer. The patient begins to resist or refuse to eat and drink, and accepts only small amounts of food with an inability to swallow [1].

### Pathology

The histological features of angiosarcoma can provide useful clues for diagnosis. Angiosarcoma can be classified into different subtypes based on the cytological appearance: spindle-shaped endothelial cells, epithelioid with large rounded or polygonal cells, and pleomorphic or mixed phenotypes as in most angiosarcomas [6]. Endothelial cells are easily identifiable in histology. However, the existence of anaplasia in most angiosarcoma cases makes it difficult to distinguish these tumors from other undifferentiated types such as hemangiopericytoma melanoma [7]. Therefore, immunohistochemistry is mandatory to establish the definitive diagnosis of angiosarcoma. Endothelium expression markers CD31, CD34, von Willebrand factor (vWF), *Ulex europaeus* agglutinin 1 (UEA-1), Friend leukemia integration 1 (Fli-1), endothelin-1, vascular endothelial growth factor receptor (VEGFR), and specific gene related erythroblastic transformations (ERG) can help identify angiosarcomas, each with different sensitivity and specificity [1, 6].

The histological classification was considered as predictive of the prognosis. Recent data suggest that the grade of angiosarcoma has no prognostic value. Low-grade lesions can metastasize. The second localizations occur in the lungs, liver, bones, and skin. Involvement of the axillary nodes is rare [7].

### Treatment and prognosis

The treatment must above all be surgical, as for the majority of sarcomas. It is imperative that the excision limits are healthy. Due to the low propensity of lymph node extension, axillary dissection is not essential. The usefulness of complementary radio-chemotherapy is controversial. No study has justified the use of hormone therapy in this setting [8].

The natural course of this lesion is toward rapid recurrence despite surgical excision. Hematogenic metastases frequently occur in the lungs, skeleton, liver, brain, and ovaries as well as in the skin [2]. Because of this potential for rapid dissemination through the blood, angiosarcoma is the breast tumor with the worst prognosis with a median survival at 2 years [8]. Angiosarcomas badly respond to chemotherapy and radiotherapy [8]. Surgery is the key treatment of the early form of this cancer although there is common recurrence [4]. An optimal adjuvant therapy is unknown, but patients receive both single-agent therapy as well as multiple-drug regimens. Because local or distant recurrence is possible despite adequate excision, adjuvant therapy may be of benefit [9]. Most authors associate the result with the size of the tumor at the time of diagnosis and the state of the margin with surgery. Median recurrence-free survival is less than 3 years [9].

Most African countries do not yet have a real cancer management policy. It is often the family’s responsibility to pay the costs of treating patients. This forces some families to opt for a traditional herbal treatment which seems to be less expensive, exposing patients to all the complications with a fatal outcome.

## Conclusion

Breast angiosarcomas are extremely rare cancers in adolescents and have a very poor prognosis. It is often a breast mass that can reach very large sizes and a diagnostic confirmation is made by immunohistochemistry. This is often fatal, especially in countries with small medical review services that do not have a policy for adequate cancer management. Breast angiosarcomas are the most formidable of the mammary tumors, and only an early surgery can bring hope for longer survival. This observation poses the problem of setting up a policy to fight breast cancer in Benin and Togo. Emphasis should be placed on awareness and early case detection.

## Data Availability

All data generated or analyzed during this study are included in this published article.
